# Implications of tyrosine phosphoproteomics in cervical carcinogenesis

**DOI:** 10.1186/1477-3163-7-2

**Published:** 2008-07-17

**Authors:** Bernice L Robinson-Bennett, James DeFord, Concepcion Diaz-Arrastia, Lyuba Levine, Hui-Qui Wang, Edward V Hannigan, John Papaconstantinou

**Affiliations:** 1 Department of Obstetrics and Gynecology, The University of Texas Medical Branch, Galveston, Texas, USA; 2 Department of Biochemistry and Molecular Biology, The University of Texas and Medical Branch, Galveston, Texas, USA; 3 Department of Histology, The University of Texas Medical Branch, Galveston, Texas, USA

## Abstract

**Background:**

Worldwide cervical cancer remains a leading cause of mortality from gynecologic malignancies. The link between cervical cancer and persistent infection with HPV has been established. At a molecular level little is known about the transition from the precancerous state to invasive cancer. To elucidate this process, cervical biopsies from human specimens were obtained from precancerous state to stage III disease.

**Methods:**

Cervical biopsies were obtained from patients with a diagnosis of cervical cancer undergoing definitive surgery or staging operation. Biopsies were obtained from patients with precancerous lesions at the time of their excisional procedure. Control samples were obtained from patients undergoing hysterectomy for benign conditions such as fibroids. Samples were subjected to proteomic profiling using two dimensional gel electrophoresis with subsequent trypsin digestion followed by MALDI-TOF protein identification. Candidate proteins were then further studied using western blotting, immunoprecipitation and immunohistochemistry.

**Results:**

Annexin A1 and DNA-PKcs were found to be differentially expressed. Phosphorylated annexin A1 was up regulated in diseased states in comparison to control and its level was strongly detected in the serum of cervical cancer patients compared to controls. DNA-PKcs was noted to be hyperphosphorylated and fragmented in cancer when compared to controls. By immunohistochemistry annexin A1 was noted in the vascular environment in cancer and certain precancerous samples.

**Conclusion:**

This study suggests a probable role for protein tyrosine phosphorylation in cervical carcinogenesis. Annexin A1 and DNA-PK cs may have synergistic effects with HPV infection. Precancerous lesions that may progress to cervical cancer may be differentiated from lesions that will not base on similar immunohistochemical profile to invasive squamous cell carcinoma.

## Introduction

Worldwide cervical cancer remains the second leading cause of cancer mortality in females [1]. The five year survival for locally advanced cervical cancer is within the range of 18% to 34% [2,3]. This poor prognosis and lack of treatment for late stage and recurrent disease probably reflects the sparse understanding of the molecular pathogenesis of disease progression. The link between cervical cancer and the Human Papilloma Virus (HPV) has long been established. However, the multi-step progression in cervical carcinogenesis from the precancerous lesions of high grade cervical intraepithelial neoplasia (high grade dysplasia) to invasive carcinoma is still to be elucidated. Unlike colon cancer which has a definite precancerous state and an elucidated model for carcinogenesis, cervical carcinogenesis with a known precancerous lesion has not yet been completely elucidated.

In general, at a molecular level cancer is considered a state of altered signaling. One of the most common mechanisms of activating and/or inactivating signaling pathways is phosphorylation and de-phosphorylation at serine, threonine and tyrosine residues. This modification controls a variety of cellular processes including cellular growth, proliferation, cell cycle control, cytoskeletal mobility and receptor regulation [4]. Phosphorylation leads to allosteric modifications that may result in sufficient conformational change to cause activation or inactivation of various proteins and associated altered functioning. We hypothesized that identification of phosphoproteins associated with the various stages of cervical cancer may provide information on the mechanism of tumorigenesis and insight in the development of diagnostic and therapeutic procedures.

The mitogen activated protein kinase (MAPK) pathways are known be deregulated in many human malignancies [5]. The best studied with regards to malignancy are the extracellular signal regulated protein kinases (ERK). ERKs phosphorylate cytoplasmic targets or migrate to the nucleus where they can activate transcription factors involved in cellular proliferation. Aberrant signaling in the MAPK/ERK has been described in prostate; breast and colon cancers in *in vitro *as well as *in vivo *model [6-8]. In cervical cancer, one study has described decreased activation of ERK1/2 in invasive cervical carcinoma [9].

Annexin A1 is a calcium dependent phospholipid binding protein that has been linked to membrane trafficking through exocytosis and endocytosis [10]. Other studies have evaluated its role in the modulation of the MAPK/ERK [11]. Many members of the Annexin family are known to undergo alternate splicing yielding a number of isoforms. The resultant variant forms may have different functions and binding capacity compared to the native forms [12].

DNA-Protein Kinase catalytic subunit (DNA-PKcs), a macromolecule found to be involved in the repair of double stranded DNA breaks through activation of p53 was found to be expressed in cancer specimens in its tyrosine phosphorylated and cleaved form [13]. In contrast, in normal specimens DNA-PKcs existed in its intact full length non-phosphorylated form.

The aim of the study was to identify differential expression and modification of proteins that could suggest aberrant pathways which could serve as novel targets for developing new therapies in the treatment of cervical cancer and in monitoring disease recurrence or progression.

## Materials and methods

### Antibodies

Annexin A1 mouse monoclonal (Santa Cruz, CA, USA), Calgranulin A8 rabbit polyclonal (Santa Cruz, CA, USA), DNA-PK catalytic subunit, multiple clones (USBiological, MA, USA). Phospho-ERK44/42 (Cell Signaling, Beverly, MA), Phospho-MEK (Cell Signaling, Beverly, MA, USA), MEK (Cell Signaling, Beverly, MA, USA), Phosphotyrosine mouse monoclonal antibody (Santa Cruz, CA, USA), p53 rabbit polyclonal antibody (Santa Cruz, CA, USA), Goat Anti-mouse secondary HRP (Alpha Diagnostics, San Antonio, TX, USA), Anti-rabbit IgG HRP secondary (Alpha Diagnostics, San Antonio, TX, USA).

### Buffers

Buffer M (100 mM HEPES-KOH, pH 7.4, 5 mM EGTA, 20 mM EDTA, 100 mM sodium orthovanadate, 0.5 M sodium fluoride, 10 mM sodium molybdate, 0.2 M β-glycerophosphate); 2-D Lysis Buffer (8 M urea, 4% CHAPS); Rehydration Buffer (8 M Urea, 2% CHAPS, 0.002% bromophenol blue, 0.75% pH 4–7 pharmalyte); Equilibration Buffer (50 mM Tris-HCL, pH 8.8, 6 M Urea, 30% of 87% Glycerol, 2% SDS, 0.002% Bromophenol blue); Transfer Buffer (25 mM Tris, 200 mM Glycine, 20% Methanol);

TBS-T (10 mM Tris pH 7.4, 154 mM sodium chloride, 0.1% Tween 20); Blocking Buffer (5% carnation nonfat dried milk or 5% BSA with phosphatase inhibitors for phosphoamino antibodies). 3× Sample Buffer (187.5 mN Tris-HCL, 6% SDS, 30% Glycerol, 150 mM DTT, 0.3% bromophenol blue).

### Patients

After obtaining IRB approval and written consents, patients were recruited from the University of Texas Medical Branch after having a biopsy proven diagnosis of high grade dysplasia (pre-cancer) or frankly invasive cervical cancer at different stages. A small biopsy was obtained from these patients. The patients' ages ranged from 16–69 years old; and the most advanced stage recruited was IIIB disease. Normal cervical tissue was obtained from women undergoing hysterectomy for benign diseases such as fibroids or endometriosis without a history of abnormal Pap smears. The study cohort consisted of normal (n = 10), precancerous (n = 10), stage I (n = 6), stage II (n = 6) and stage III (n = 5).

### Tissue processing

Biopsy specimens were collected in a commercially prepared solution of RNA *later *(Ambion, Austin, TX, USA) in the operating room and then stored at -80°C until further processing. The specimens were subsequently rinsed in PBS, minced in Buffer M and homogenized in the same medium using a polytron at full speed and subsequently centrifuged at 13,000 rpm for 10 minutes. Protein quantification was performed on the supernatant using the Bradford Assay (BioRad reagent). Aliquots of 100 μg and 200 μg were then stored at -80°C to minimize the effects of tissue freeze thawing. In each analysis individual patient samples were used and not pooled.

### Two dimensional gel electrophoresis (2D-GE) and MS sequencing and 1D SDS-PAGE

Aliquots of cell lysates (200 μg) were diluted with rehydration buffer and 3.2 μl of 1 M DTT. pH 4–7 IPG immobiline dry strips were rehydrated with 200 μl of a combined mixture of sample at 20°C for 12 hours. Iso-electric focusing was performed for a total of 18000 Vh. IPG strips were subsequently incubated in equilibration buffer at room temperature on an orbital shaker for 15 minutes. Samples were then separated by SDS-PAGE in the second dimension on 10–20% Tris-Glycine BioRad precast gels.

Gels prepared for Matrix Laser Desorption Ionization – Time of Flight Mass Spectrometry (MALDI-TOF MS) were stained using GelCode Blue Stain Reagent from Pierce Biotechnology. The gel pieces were then trypsin digested and proteins were identified by MS fingerprinting of peptides using ProFound data search.

### Immunoprecipitation and co-immunoprecipitation

Aliquot of cell lysates (200 μg) from normal, pre-cancerous and cancerous specimens were incubated with Protein G Sepharose beads (Sigma-Aldrich,) for 2 hours at 4°C for pre-clearing. Beads and protein mixtures were then centrifuged at 13,000 rpm for 1 minute. Aliquots of 4 μl antibody were added to supernatant in fresh microfuge tube and incubated with constant rotation for 4 hours at 4°C. Fresh beads were then added and the mixture was allowed to rotate overnight at 4°C. The beads were then collected after centrifugation and were washed three times with 500 μl of PBS. 100 μl of 3× sample buffer was added to immunopellets which were then boiled for 5 minutes. Mixtures were centrifuged at 13,000 rpm for 2 minutes and the supernatant collected. 15 μl aliquots of the supernatant were then subjected to separation by SDS-PAGE on 5% or 4–20% Tris-Glycine precast BioRad gels.

### Western blotting

Tissue lysates and immunoprecipitates were prepared as described above and subsequently transferred to PVDF membranes. Membranes were blocked for 1 hour in 5% non-fat dry milk in 0.1% Tween 20 in TBS (pH7.4). Primary antibodies were diluted in 5% nonfat dry milk, 0.1% Tween 20 in TBS and incubated at 4°C overnight. Membranes were washed in 0.1% Tween 20 in TBS. Secondary horseradish peroxidase (HRP) antibody was diluted 1:4000 in non fat dry milk in 0.1% Tween 20 and incubated for 1 hour at room temperature. Membranes were washed and incubated with HRP chemiluminescence reagents from Millipore (Billerica, MA, USA) and developed after exposure to Biomax ML film. Loading controls were derived from stained Coomasie gels.

### Immunohistochemical analysis

Buffered formalin-fixed, paraffin-embedded tissue sections (5 μm) were deparaffinized and rehydrated by passage through xylene and graded ethanol solutions. Slides were then treated with 3% hydrogen peroxidase with 0.03% sodium azide in PBS for 10 min, followed by microwave antigen retrieval at 100°C for 10 min in DAKO Target Retrieval Solution (DAKO Corporation, Carpinteria, CA) in a H2800 Microwave Processor (Energy Beam Sciences, Inc; Agawam, MA). Slides were incubated in 0.05% casein (Sigma, St. Louis, MO)/0.05% Tween-20 (DAKO Corporation, Carpinteria, CA)/PBS for 30 min to block nonspecific protein binding. Mouse monoclonal antibody, Annexin AI (Santa Cruz Biotechnology Inc, Santa Cruz, CA) was applied to sections at a 1:400 dilution for 60 min. Mouse IgG Ready-To-Use (InnoGenex, San Ramon, CA) was used as a negative control. DAKO EnVision+ System-HRP Labeled Polymer Anti-mouse (DAKO Corporation, Carpinteria, CA) served as the detection system, and colorized by DAB (DAKO Corporation, Carpinteria, CA). Slides were counter-stained with Mayer's Modified Hematoxylin (Poly Scientific, Bay Shore, NY) before mounting, viewed under an Olympus BX51 microscope and images recorded by DP70 Digital Camera (Olympus Optical Co., Ltd; Tokyo, Japan).

### PF2D serum fractionation and western blotting

Protein fractionation 2D profiling is a HPLC system developed by Beckman which separates proteins based on two dimensional HPLC. First proteins are separated by their iso-electric points in the first dimension. Fractions were then collected in 96 well plates and are re-injected into a second dimension reverse phase column and separated based on hydrophobicity and again collected in 96 well plates.

Whole blood was centrifuged at 3,000 × *g *and 100 μl aliquots of serum were buffer exchanged in Beckman start buffer (proprietary). Protein quantification was done using the Bicinchoninic assay and 2–5 mg of serum was injected in Beckman PF2D HPLC System for fractionation. Fractions were collected in 96 well plates in the first and second dimension. Proteins were then transferred to PVDF membranes via the Biomek 2000 robot and a Schleicher & Schuell dot blotting apparatus and subsequent western blotting performed in the manner described above.

## Results

### Identification of annexinA1 by 2DGE/MALDI-TOF with validation by immunoblotting

**Figure 1:**
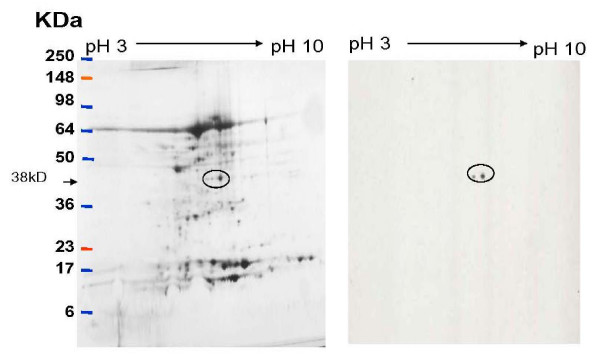
Characteristic two dimensional gel demonstrating protein expression in cervical cancer form tissue lysate. The left panel shows the entire proteome in a stage II cancer specimen. Western blotting with monoclonal annexin A1 showed two distinct spots as depicted in panel B.

Annexin A1 was identified by MALDI-TOF MS after in gel trypsin digestion to be differentially expressed in cancer samples compared to controls when 2D gels were compared. Once this candidate protein was found then its presence was validated by western blotting (Figure 1).

### 32 kD annexin A1 is differentially tyrosine phosphorylated in cervical cancer compared to normal

**Figure 2:**
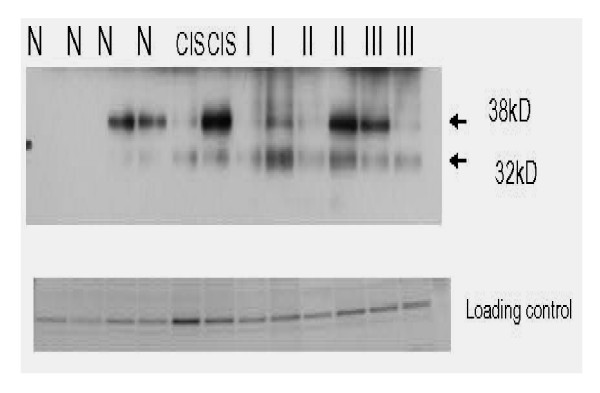
Expression of annexin A1 with disease progression. Immunoprecipitation with phosphotyrosine antibody followed by one dimensional SDS-PAGE and western blot with annexin A1 antibody. Two phosphorylated isoforms noted. 32 kD form correlates well with disease progression. (N = normal, CIS = pre-cancer or high grade dysplasia, I = stage I, II = stage II, III = stage III).

To determine if the annexin A1 expressed in the cancerous state was phosphorylated, immunoprecipitation with phosphotyrosine antibody was carried out followed by western blotting with annexin A1 monoclonal antibody. 38 kD and 32 kD bands were identified. There was an obvious up-regulation of the modified 32 kD protein noted with disease progression. The expression of the 38 kD phosphotyrosine form was less specific for disease progression (Figure 2). The reverse immunoprecipitation was carried out with annexin followed by western blotting with phosphotyrosine antibody and similar results were obtained (data not shown).

### Differential expression and localization of Annexin A1 in cancer specimens compared to controls

**Figure 3:**
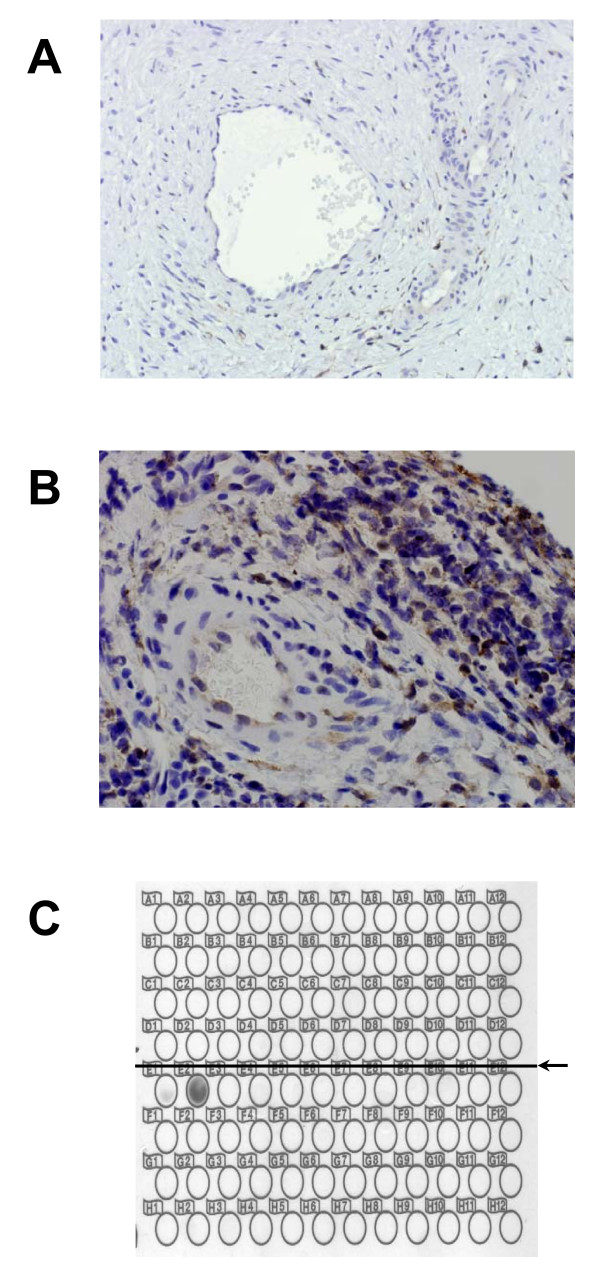
**Immunohistochemical analysis of paraffin embedded slides (×400)**. (A) Absent staining of microvasculature endothelium in normal biopsy specimen. (B) Positive staining of endothelial cells lining microvasculature in cervical cancer specimen. (B) (C) Representative PF2D HPLC analysis followed by western blot with annexinA1 monoclonal antibody of serum from control patient (upper panel) demonstrating absence of annexinA1 compared to presence of annexinA1 in serum of patient with stage II cervical cancer (lower panel).

The endothelial lining of the vascular spaces in cancer tissues exhibited annexinA1 staining in contrast to weak or absent staining in control samples (Figure 3A and 3B). Complementary to this was the observation that by chromatographic separation using the Beckman PF2D Proteome Lab annexin A1 was strongly expressed in the sera of cervical cancer patients and was absent in sera of a cohort of unexposed aging nuns (Figure 3C). With prolonged exposure of the film, weak staining was observed in the sera of some patients with pre-invasive disease (data not shown).

### Aberration in MAPK/ERK pathway with disease progression and co-migration with annexin A1

**Figure 4:**
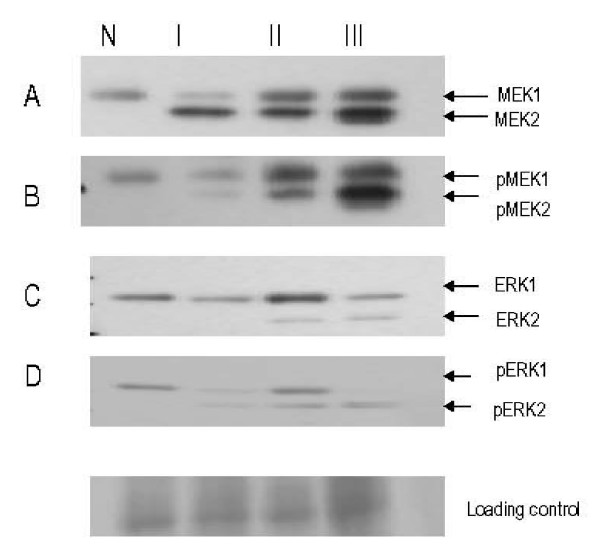
**Expression of MAPK/MEK and MAPK/ERK with cervical cancer progression by western blotting**. Tissue lysates were subjected to SDS-PAGE followed by western analysis with MEK1/2, pMEK1/2, ERK1/2 and pERK1/2. Phosphorylated forms of MEK1/2 and ERK1/2 are upregulated in cancer specimens. MEK2 and ERK2 are consistently not expressed in normal specimens.

Previous studies have shown that annexin A1 specifically target the MAPK/ERK pathway at a site upstream of MEK1/2 [11]. Therefore, we sought to investigate the expression of ERK1/2 with disease progression and to analyze if its expression would be linked to annexin A1 expression. Total ERK1/2 and MEK1/2 demonstrated up-regulation with advancing disease stage. Of interest MEK2 and ERK2 were consistently absent in the normal samples studied. The phosphorylated activated forms of MEK 1/2 and ERK 1/2 showed a stronger fold change in expression with advancing disease (Fig. 4). Additionally, annexin A1 was shown to be in complex with activated pERK and pMEK (data not shown).

### Validation of DNA-PKcs and differential tyrosine phosphorylation with advancing disease

**Figure 5:**
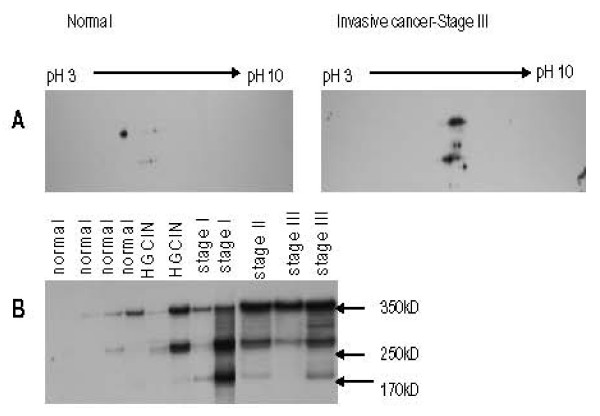
**DNA-PKcs expression by immunoprecipitation and western blot analysis**. (A) Differential expression of DNA-PKcs in cancer compared to normal. Demonstrated by anti-DNA-PKcs antibody 2DGE suggests a macromolecule with multiple fragments and shift in iso-electric point in cancerous samples. (B) Immunoprecipitation with phosphotyrosine antibody followed by western blot with DNA-PKcs, showing fragmentation of molecule as early as high grade precancerous lesion.

Phosphotyrosine immunoprecipitate was analyzed by 2DE. In gel digestion of candidate spot identified DNA-PKcs as a modified protein. Western blot analyses on 2DE using mouse monoclonal DNA-PKcs specific antibody confirmed the presence of a large multi-subunit protein. The pattern of resolution by iso-electric points was different in cancer compared to normal (Figure 5A). The 350 kD full length protein detected in normal and precancerous specimen was not significantly phosphorylated. In contrast, in invasive cancer tissues, the full length protein as well as a 250 kD and 170 kD fragments were consistently detected as being hyperphosphorylated at tyrosine residues (Figure 5B). By immunoprecipitation and western blot we demonstrated that in cervical cancer specimens DNA-PKcs failed to bind to p53 (data not shown).

### Calcium binding protein and tyrosine modification in cervical cancer

**Figure 6:**
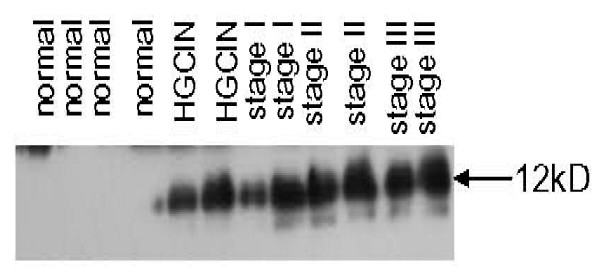
Differential expression of tyrosine modified calcium binding protein, calgranulin (S100A8). As demonstrated by western blotting tyrosine phosphorylated calgranulin A8 demonstrated differential expression with disease progression as early as the precancerous stage of cervical carcinogenesis.

By 2DGE multiple S100 calcium binding proteins were shown to be modified by tyrosine phosphorylation, in particular S100A8 and S100A9. After identification by MALDI-TOF, tissues were then subjected to SDS-PAGE and western blotting with specific antibodies. Calgranulin A8 did show differential expression with disease progression. Consistently, the protein was identified in its unmodified form in normal tissue. However, even at the precancerous states the protein was noted to be tyrosine phosphorylated (Figure 6).

## Discussion

While the incidence of abnormal Pap smears and dysplasia is very high, only 1–2% of true precancerous lesions will progress to invasive cancer. The issue that begs to be addressed is the distinguishing feature(s) between the lesions that will progress and those that will not. The aim of our study was to gain a more complete understanding of cervical tumorigenesis at the molecular level using tyrosine phosphoproteomics. We hypothesize that patients with high grade dysplastic lesions exhibiting annexinA1 in the serum and tumor vasculature will be at risk for disease progression since the identical pattern is seen in invasive cancer. This theoretically could serve to differentiate the true pre-cancerous lesions with potential of progressing to invasive cervical cancer from those that will not.

Annexin A1 is a dual calcium and phospholipid binding protein which has been implicated in inflammation and numerous human cancers, including head and neck, pituitary and prostate cancers [14,15]. It belongs to a family of twenty such proteins with high structural homology in the 70 amino acid repeat sequence. The multifunctional properties and specificity of the molecule are mediated through its N-terminal domain. Annexin A1 has been shown to play critical roles in membrane trafficking through endocytosis and exocytosis [10] as well as serve as a scaffold protein in multi-protein complexes. It has also been shown to be a major substrate for EGFR kinase [16]. Alldridge *et al *showed that annexin A1 regulates the MAPK/ERK pathway [11]. The MAPK/ERK when activated through phosphorylation is noted for its role in cell proliferation. Tyrosine phosphorylation of annexin A1 has been observed in pituitary carcinomas but not adenomas [14]. Recently, annexin A1 translocation was noted in esophageal squamous cell carcinoma [17]. In our study we have identified two forms of annexin A1. The 32 kD form was not detected on the 2DGE analysis but was seen clearly on the immunoprecipitation followed by western blot (Figure 1 and Figure 2). This is not unexpected as it is known that immunoprecipitation significantly concentrate proteins present in whole lysates compared to 2DGE. There is a trend toward upregulation of the 32 kD form of the protein in disease states including precancerous states compared to normal and seems to be more specific than the 38 kD form which demonstrated heterogeneity. Smith *et al *demonstrated that in human lung lavage fluid, annexin A1 presented as a doublet (37/33 kD) by western blotting. In that study, annexin A1 in neutrophils was the cleaved 33 kd form while the intact 37 kD form was recovered from the cell surface [18]. In human model of skin inflammation the 33 kD form was prevalent [19]. Additionally, previous studies have shown that phosphorylation of annexin A1 makes it susceptible to proteolytic degradation [20] and phosphorylation of the 32 kD form results in its exocytosis [21]. Further studies by Wang *et al *have shown that phosphorylation at the N-terminal decreases association of annexin A1 to plasma membrane because of its decreased affinity for calcium binding and therefore requiring larger internal calcium concentrations [22]. Additionally, in our study a number of calcium binding proteins were found to be phosphorylated in precancerous specimens and invasive cervical cancer specimens.

Previous studies have shown that annexin A1 antibody has been found in the serum of patients with lung cancer [23]. We have shown through liquid chromatography that annexin A1 is present in the serum of patients with invasive cervical cancer and certain precancerous specimens. Additionally, by IHC the endothelial cells lining the vasculature in the microenvironment in cancer stained positive for annexin A1 but absent staining was noted in the controls. We propose that in cervical dysplasia/pre-cancer and invasive cancer, annexin A1 tyrosine phosphorylation leads to its solubilization and subsequent extravasation in serum as evidenced by its presence in tumor microvasculature but not in microvascular environment in controls. In addition, alteration in the function, probable loss of tumor suppressor role of the native protein may occur as a result of the phosphorylation. Tyrosine phosphorylation of annexin A1 may be essential in the malignant transformation of squamous epithelium of the cervix. While this expression is not specific to cervical cancer, in a patient with no other underlying pathology annexin A1 expression in the serum maybe used as a marker for disease progression, recurrence or to monitor response to therapy. The mechanism of secretion remains unclear but may partially be due to its phosphorylation and subsequent cleavage. It has been reported that extracellular annexin A1 through its attachment to formyl peptide receptors may lead to inhibition of neutrophil extravasation to sites of inflammation [24]. This could potentially explain a means whereby HPV escape immune surveillance leading to persistent disease and ultimately invasive cervical cancer.

Consistent with its role as a scaffold protein we have shown annexin A1 to be in complex with MEK1/2, ERK1/2. There was no difference in the expression of MEK1 or ERK1 with disease progression; however, there was a marked up-regulation in the activated forms of ERK2 and its immediate upstream activator, MEK2 with disease progression. This pathway may be affected by the up-regulation of phosphorylated annexin A1 and may play a role in the carcinogenesis of cervical cancer through increase cellular proliferation.

The link between annexin A1 and cervical cancer may have clinical significance. In a recent study, annexin A1 antibody given to tumor bearing rats with advanced lung cancer improved survival and decrease weight loss [25]. Based on our observation, annexin A1 could potentially serve as a target to image, monitor and treat cervical cancer, particularly in advance or recurrent cases where therapeutic options are presently limited.

Another protein that was noted to be modified by tyrosine phosphorylation in cancer as well as in precancerous states and also shown to co-immunoprecipitate with annexin A1 is DNA-PKcs. In the presence of double stranded DNA breaks (DSB), activated DNA-PKcs is responsible for the activation of p53 through binding and serine phosphorylation. Previous studies have shown that DNA-PKcs may be inactivated by tyrosine phosphorylation [26,27]. Other studies have demonstrated that inactivation of DNA-PKcs results in hyperplasia, dysplasia of the intestinal mucosa in colon cancer [13]. HPV E6 viral oncoprotein has been shown to bind to p53 and target it for degradation by ubiquitination. In our study we have demonstrated that DNA-PKcs has been modified by tyrosine phosphorylation in diseased states, coupled with its fragmentation and hence presumed inactivation. This therefore could represent a novel way by which p53 is inactivated in cervical carcinogenesis and may be synergistic with the well known inactivation mechanism through the HPV E6 viral oncoprotein. Previous studies have shown fragmentation and inactivation of DNA-PKcs by polio virus or adenovirus but none have linked its inactivation to HPV [28-31]. Further studies are therefore needed to elucidate any interaction between HPV and DNA-PKcs.

While our sample size may be insufficient to perform power analyses, the trends observed deserves consideration and additional studies. Potential biomarkers may be present amongst these modified proteins as well as immunotherapeutic and dietary supplemental agents to treat the disease in a cancer with very limited therapy in the recurrent and advanced disease. Future studies will be aimed at investigating phosphorylation at serine and threonine sites in diseases states and control in an effort to mine the phosphoproteome of cervical carcinogenesis.

## Conclusion

In this study we have demonstrated that there is a trend toward upregulation of the tyrosine modified proteins, Annexin A1, DNA-PKcs and certain calcium binding proteins in cancer specimen in contrast to normal. We have also demonstrated that a subset of patients with precancerous disease demonstrate similar protein expression and modification as in the invasive cancer. Based on the data provided it may be feasible to distinguish precancerous lesions with true invasive potential from those that are less likely to progress to invasion. Annexin A1 expression.

## Competing interests

The authors declare that they have no competing interests.
